# Transplantation of placenta‐derived mesenchymal stem cells enhances angiogenesis after ischemic limb injury in mice

**DOI:** 10.1111/jcmm.12489

**Published:** 2015-08-18

**Authors:** Nanzi Xie, Zhihong Li, Timothy M Adesanya, Weixin Guo, Yang Liu, Minghuan Fu, Ahmet Kilic, Tao Tan, Hua Zhu, Xiaoyun Xie

**Affiliations:** ^1^ Division of GeriatricsTongji HospitalTongji UniversitySchool of MedicineShanghaiChina; ^2^ Division of General SurgeryChenzhou First People's HospitalChenzhouHunanChina; ^3^ Department of SurgeryDavis Heart and Lung Research InstituteThe Ohio State University Wexner Medical CenterColumbusOHUSA; ^4^ Guangdong Geriatrics InstituteGuangdong General HospitalGuangdong Academy of Medical SciencesGuangzhouChina

**Keywords:** placenta, stem cells, angiogenesis, ischemia, paracrine

## Abstract

Mesenchymal stem cell‐based therapy has emerged as a promising approach for the treatment of peripheral arterial disease. The purpose of this study was to examine the potential effects of human placenta‐derived mesenchymal stem cells (PMSCs) on mouse hindlimb ischemia. PMSCs were isolated from human placenta tissue and characterized by flow cytometry. An *in vivo* surgical ligation‐induced murine limb ischemia model was generated with fluorescent dye (CM‐DiI) labelled PMSCs delivered *via* intramuscular injection. Our data show that PMSCs treatment significantly enhanced microvessel density, improved blood perfusion and diminished pathologies in ischemic mouse hindlimbs as compared to those in the control group. Further immunostaining studies suggested that injected PMSCs can incorporate into the vasculature and differentiate into endothelial and smooth muscle cells to enhance angiogenesis in ischemic hind limbs. This may in part explain the beneficial effects of PMSCs treatment. Taken together, we found that PMSCs treatment might be an effective treatment modality for treatment of ischemia‐induced injury to mouse hind limbs by enhancement of angiogenesis.

## Introduction

Peripheral arterial disease (PAD) is a major global health problem, especially with the ageing mean population in developed countries 1. PAD refers to a set of disorders in which progressive stenosis and ultimately occlusion of peripheral arteries results in impaired blood flow to the lower limbs. In the late stages of PAD, progression of tissue hypoperfusion results in ischemic ulceration. Unfortunately, amputation may be necessary in approximately 30% of these patients and prognosis following amputation is bleak with a mere 30% 5‐year survival 2. Treatment in the hopes of limb salvage often requires invasive revascularization procedures, which may not be suitable for a significant percentage of patients with prohibitive co‐morbidities. Therapies designed at improved perfusion, namely angiogenesis, will be essential for the restoration of tissue function with the hopes of reducing the need for limb amputation. A need for new therapeutic strategies that are less invasive and available for the entire population of patients is apparent.

Angiogenesis continues to be a futile ground for investigation for the treatment of PAD. Local administration of angiogenic genes or small molecules have shown promise in preclinical studies, but in randomized clinical trials, improvement has been modest and transient at best 3. Recently, efforts have shifted to stem cell‐based approaches because of the theoretical capacity of stem cells to differentiate and form new blood vessels in a directed fashion 4, 5. In particular, bone marrow‐derived MSCs (BM‐MSCs) and endothelial progenitor cells (EPCs) have undergone the most translational and human studies among all the various stem cell approaches 6. However, there are challenges in the use of BM‐MSCs and EPCs 2, 7. The survival and differentiation potential of BM‐MSCs or EPCs obtained from aged patients and/or with age‐related disorders limit their therapeutic efficiency. Furthermore, autologous delivery of cells inevitably means a delay in treatment due to the time needed to collect, isolate, and propagate the cells to obtain adequate numbers for injection 8. These limitations have necessitated investigation of other sources for stem cell therapy.

In our present study, we isolated a population of mesenchymal stem cells (MSCs) from the human term placenta. These placental mesenchymal stem cells (PMSCs) display typical mesenchymal characteristics, such as, multipotent differentiation and expression of surface antigens similar to those of BM‐MSCs. We therefore hypothesized that PMSCs administration would restore blood perfusion in a model of PAD. We evaluated the effect of PMSCs transplantation on blood flow in a murine model of hindlimb ischemia. In addition, we also sought to elucidate the mechanisms of their action by analysing their capacity to differentiate into endothelial and smooth muscle cells.

## Materials and methods

### Materials

Lectine from *Ulex europaeus* fluorescein isothiocyanate (FITC) conjuate (FITC‐UEA‐1), trypsin‐EDTA, bovine serum albumin, human fibronectin (FN), bFGF, penicillin, streptomycin, VEGF, hFGF, and heparin were obtained from Sigma‐Aldrich (St. Louis, MO, USA). CM‐DiI dye, DMEM, foetal bovine serum (FBS), L‐glutamine, and nonessential amino acids were purchased from Invitrogen Corporation (Gibco, Grand Island, NY, USA). CD29 [allophycocyanin (APC)‐conjugated], CD13 (FITC‐conjugated), CD73 [phycoerythrin (PE)‐conjugated], CD105 (PE‐conjugated), CD49b (APC‐conjugated), HLA‐DR (FITC‐conjugated), CD45 (PE‐conjugated), and CD34 (FITC‐conjugated) antibodies were obtained from Becton, Dickinson and Company (BD Pharmingen, San Diego, CA, USA). Adipogenic, chondrogenic, and osteogenic differentiation from PMSCs were evaluated in the appropriate induction media, also obtained from Invitrogen. Anti‐vWF, anti‐CD31, anti‐CD45, anti‐human α‐SMA and anti‐Bcl‐xl antibodies for second antibody (2nd Ab) incubations were obtained from Santa Cruz Biotechnology (Santa Cruz, CA, USA). *In vitro* angiogenic analysis of differentiated endothelial‐like cells was performed using the *in vitro* angiogenesis kit obtained from Chemicon International, Inc. (Temecula, CA, USA). Quantitative PCR amplification and detection were performed with SYBR Premix Ex Taq (TaKaRa, Shiga, Japan). (DiI)‐labelled acetylated low‐density lipoprotein (DiI‐AcLDL) was measured to determine endothelial cells (Molecular Probes, Milano, Italy). Unless otherwise specified, all other reagents were purchased from Sigma‐Aldrich.

### Patient selection, tissue processing and MSC isolation

Placental MSCs isolation protocol was established from our previous study 9. Briefly, resh placentas were collected from normal, full‐term (38–40 weeks gestation), healthy donors in compliance and approval of the Independent Ethics Committee of the Tongji Hospital affiliated with Tongji University. Written informed consent was signed prior to the study. Before the placentas were dissected, umbilical cord blood was allowed to drain. All tissues were examined by a certified pathologist to exclude human immunodeficiency virus, toxoplasmosis, cytomegalovirus and rubella virus infections. To preserve cell viability, all tissues were processed within three hours after the pathologist evaluation.

The harvested tissues were washed four times in PBS and then manually minced and digested with 0.1% collagenase IV (Sigma‐Aldrich) at 37°C for 1 hr. The tissue was filtered twice through a cell strainer (Falcon 3078; BD Biosciences, San Jose, CA, USA) to eliminate undigested fragments. After the cells were centrifuged at 350 g for 10 min., and red blood cells were lysed by red blood cell lysis buffer for 5 min. at 37°C, then the remaining cells were centrifuged at 300 *g* for 5 min. The cell pellets were resuspended in DMEM medium containing 10% FBS, 100 units/ml penicillin, 100 μg/ml streptomycin, 2 mM L‐glutamine, and 1% nonessential amino acids. The cells were cultured at 37°C under a 5% CO_2_ atmosphere for 4 days before the culture medium was first changed and a 70–80% cellular confluence was obtained.

### Determination of angiogenic factors in PMSCs media cultured in normal and hypoxic conditions

Placental MSCs were cultured under hypoxic conditions (2% O_2_) for up to 72 hrs. A growth factor‐free endothelial cell basal medium‐2 (Lonza, Ltd., Basel, Switzerland) with 1% FBS was employed in this step and served as a control. After incubation, the culture supernatant was collected, sterile filtered, and stored at −80°C until use. The hypoxic‐conditioned media of the PMSCs were analysed for the angiogenic growth factor VEGF with an ELISA kit (Pierce Biotechnology, Thermo Fisher Scientific, Rockford, IL, USA). Data were expressed as mean ± SEM picograms of the secreted factor per 10^6^ cells at the time of harvest.

### Animal studies

All animals were treated and procedures performed in accordance with the guidelines published by the Shanghai Experimental Animal Center of the Chinese Academy of Sciences, and the experiments conformed to the *Guide for the Care and Use of Laboratory Animals*.

### Hindlimb ischemia model

We evaluated the endothelial potential and neovascularization capacity of PMSCs using a murine model of hindlimb ischemia. All procedures were performed on male C57BL/6J mice (10–12 weeks, 20–25 g, *n* = 15 pairs for blood flow studies, *n* = 3 pairs for cell tracking studies). Mice were anesthetized with 50 mg/kg sodium pentobarbital via intraperitoneal injection. The left femoral artery, great saphenous artery, iliac circumflex artery/vein, and muscular branch were ligated to induce left hind limb ischemia.

### Delivery of PMSCs into C57BL/6J mice

Placental MSCs at passage 3 were marked with the fluorescent CM‐DiI dye (Molecular Probes) before cellular transplantation. One day post procedure, the ischemic limb received an intramuscular injection of 5 × 10^5^ PMSCs at four different sites, as described in previous study 10. The control group was injected similarly, but with PBS. Animals were sacrificed at different time points, and the treated muscles were harvested and cryopreserved in optimal cutting temperature (OCT) media compound (Bio‐Optica, Milan, Italy). Frozen tissue sections were used for histological evaluation of cell distribution. FITC‐UEA‐1 was used to detect the differentiated PMSCs.

### Analysis of angiogenesis

Tissue sections from the lower muscles of ischemic and healthy limbs were harvested on days 7, 14, 21 and 28 post‐procedure. Tissue samples from the healthy hindlimbs were also examined for incorporation of PMSCs. After CM‐DiI‐labelled PMSC transplantation, mice were euthanized with an overdose of pentobarbital, and ischemic tissue was obtained. Multiple frozen sections of 6 mm‐thick tissue were prepared and examined under fluorescence microscopy.

Blood flow restoration in ischemic limbs after treatment was evaluated by laser Doppler imaging (Moor Instruments, Wilmington, Delaware, USA). The animals were anesthetized with 0.25% tribromoethanol, placed on a heating pad at 37°C, and warmed to a core temperature of 37°C. Regions of interest on the ischemic or non‐ischemic hindlimb were drawn in a standard fashion. The level of perfusion in the ischemic and normal hindlimbs was quantified using the mean pixel value within the region of interest, and the relative changes in hindlimb blood flow were expressed as the ratio of the left hindlimb (ischemic) over the right hindlimb (normal). Measurements were performed on days 7, 14, 21, and 28 post‐procedure 3.

### Western blot analysis

Seventy‐two hours after ischemia injury, the Tibialis anterior (TA) muscle was isolated and lysed by Radioimmunoprecipitation assay (RIPA) buffer [25 mM Tris‐HCl (pH 7.6), 150 mM NaCl, 1% NP‐40, 1% sodium deoxycholate, 0.1% SDS] supplemented with protease inhibitor cocktail (Roche, Applied Science, Basel, Switzerland). For each sample, 25 μg of total protein was loaded and separated on 8% SDS polyacrylamide gels. Then proteins were transferred to polyvinylidene difluoride membrane and blotted with primary antibody and horseradish peroxidase conjugated secondary antibody. Peroxidase activity was developed with ECL kits (Pierce). Anti‐Bcl‐xl and anti‐GAPDH antibodies were used in the experiments.

### Histology

The mice were euthanized, and hindlimb muscles were removed and immediately frozen in OCT compound and cryosectioned for histological and immunofluorescent assessment. Representative tissue sections were processed for routine hematoxylin and eosin staining.

### Immunostaining assays

Mouse tissues were fixed with paraformaldehyde, embedded in paraffin, and sectioned for standard immunostaining. To quantitatively study fibrosis, inflammation, and vascular differentiation, tissues were stained with Masson's trichrome, rat anti‐mouse CD45 (Sigma‐Aldrich), and monoclonal mouse anti‐human CD31(Sigma‐Aldrich), respectively. The extent of neovascularization was assessed by measuring capillary density. Capillary density was quantified, using ImageJ software (NIH, Bethesda, MD, USA) to count the number of CD31 positive capillaries and then average the six sections from each sample to calculate the average positive immunoactivity 11, 12.

### Statistical analysis

Data are generally expressed as means ± SD. SPSS software version 11.0 (SPSS, Inc., Chicago, IL, USA) was used for statistical analyses. Statistical significance among mean values was evaluated using a least significant difference *t*‐test for comparison. The comparative incidence of limb salvage was evaluated by chi‐squared. Probability values of *P* < 0.05 were considered to indicate statistical significance.

## Results

### Characterization of PMSCs

**Figure 1:**
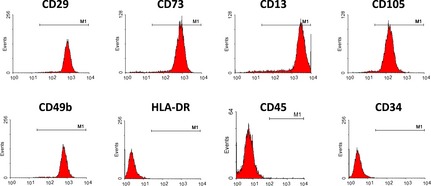
Characterization of PMSCs by flow cytometry. Most of the PMSCs strongly expressed CD29, CD73, CD13, CD105 and CD49b but were negative for HLA‐DR, CD45, and CD34. The immunophenotype of PMSCs remained unchanged for more than eight cell passages.

To characterize the phenotypes of PMSCs, flow cytometry was performed to analyse of surface markers of PMSCs. Cells were labelled with FITC‐, APC‐, or PE‐conjugated antibodies and examined by flow cytometry. Cells were dissociated and stained with CD29 (mononuclear cell marker), CD73 (endothelial cell and stem‐cell marker), CD13 (mesenchymal stem‐cell marker), CD105 (mesenchymal stem‐cell marker), CD49b (mesenchymal stem‐cell marker), HLA‐DR (an MHC class II cell‐surface receptor encoded by the human leukocyte antigen; DR is also a marker for immune stimulation), CD45, and CD34. Stained cells and an unstained control cells were subjected to FACS analysis. Most of the PMSCs strongly expressed CD29, CD13, CD73, and CD105, CD49b and were negative for HLA‐DR, CD45, and CD34 (Fig. 1). The immunophenotype of PMSCs remained unchanged for more than eight cell passages.

### *In vitro* differentiation of PMSCs into endothelial cells and secretion of angiogenesis‐related cytokines from PMSCs

**Figure 2:**
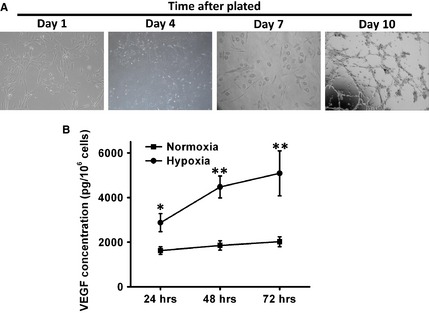
PMSCs undergo *in vitro* differentiation into endothelial cells. (**A**) Morphological changes and characterization of endothelial cell differentiation from PMSCs during the 10‐day procedure. PMSCs were plated on Matrigel‐coated culture dishes on the first day. Four days after plating, adherent cells with shorter spindle appeared. Spindle‐shaped cells were formed in 7 days. Numerous spindle‐shaped cells appeared after 10 days in culture. (**B**) Secretion of VEGF by PMSCs cultured in normal or hypoxic conditions at 24, 48, 72 hrs was measured by ELISA and is presented as mean ± SEM picograms of secreted factor normalized to 10^6^ cells at the time of harvest. Growth factor production in normal and hypoxic conditions was compared using a paired *t*‐test (*P < 0.05, **P < 0.01).

It has also been suggested that different culture conditions may differentially impact the transcriptome, proteome, and cellular architecture of MSCs. MSCs are able to differentiate into functional cells—not only mesoderm but also endoderm 13. PMSCs exhibit multilineal differentiation ability. To test whether PMSCs can differentiate into endothelial cells, we successfully modified a protocol that had been used to induce umbilical cord stem cells into endothelial cells 3, 14 and formulated a 10‐day differentiation protocol for the conversion of endothelial cells 9 (Fig. 2A).

Mesenchymal stem cells are known to secrete multiple angiogenic growth factors, such as VEGF, at levels that are bioactive 15, 16. The relative contribution of a paracrine effect of PMSCs transplantation on hindlimb ischemia was investigated. Supernatants of PMSCs in 1% FBS culture medium were harvested after exposure to hypoxic conditions (2% O_2_) for 72 hrs. Many shared characteristics and differences were observed in the cytokine. VEGF was detected in both groups and are important for cell survival and differentiation. As shown in Figure 2B, the level of VEGF was significantly higher under hypoxic condition at different time points (24 [*P* < 0.05], 48 [*P* < 0.01] and 72 [*P* < 0.01] hrs in hypoxic condition).

### PMSCs transplantation improves recovery of ischemia‐induced injury to mouse hindlimbs

**Figure 3:**
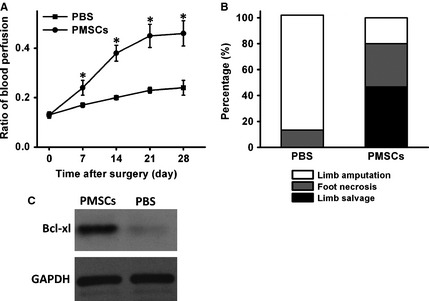
PMSCs transplantation induces *in vivo* angiogenesis in a hindlimb ischemia model. (**A**) Hindlimb ischemia was induced by femoral artery ligation in the mice, and animals were treated with either PBS or PMSCs 1 day after ligation. Quantitative analysis with laser Doppler was performed, and data are presented as a ratio of perfusion in the ischemic left leg compared with the nonischemic right leg over time on days 7, 14, 21, and 28. Data were presented by mean ± SD (*P < 0.05). (**B**) At day 21 after treatment, the comparison between the PMSCs group and the PBS group showed a significant difference in physiological status of ischemic limbs rated in three categories: limb salvage, foot necrosis, and limb loss.

In order to test the *in vivo* function of PMSCs in ischemic hindlimb injury, the PMSCs were freshly isolated from healthy donors and characterized by our established methods with detection of surface markers by flow cytometry (Fig. 1) 9. Then, PMSCs were implanted following surgery‐induced ischemia with PBS treatment being used as control. Blood flow was measured by laser Doppler imaging at different time points. Notable improvement in hindlimb mean perfusion ratio in the PMSC treatment group was observed in comparison with the control group (Fig. 3A). Furthermore, the PMSC group showed an improvement in physiological status of ischemic limbs after 21 days of transplantation as compared to the control group (Fig. 3B). PMSC transplantation was associated with successful limb salvage in 7 of 15 (46.7%) animals at day 21 after treatment. Foot necrosis was limited to five mice (33.3%), and only three experienced spontaneous limb amputation (20.0%). In contrast, of 15 mice receiving PBS injection for hindlimb ischemia, 13 of them (88.7%) suffered limb loss and 2 (13.3%) demonstrated moderate to severe necrosis from toe to knee. To determine the molecular mechanism underlying beneficial effects of PMSCs, the injured hind limb tissues were subjected to western blot analysis, as shown in Figure 3C, PMSCs treatment led to greatly enhancement of anti‐apoptotic protein, Bcl‐xl, indicating a role for PMSCs in regulating apoptosis following ischemia limb injury. These results suggest that PMSC delivery enhances the restoration of blood perfusion following ischemic injury and facilitated recovery of the injured hind limb through regulating apoptosis signalling following ischemic limb injury.

### PMSCs treatment improves pathologies associated with hindlimb ischemia

**Figure 4:**
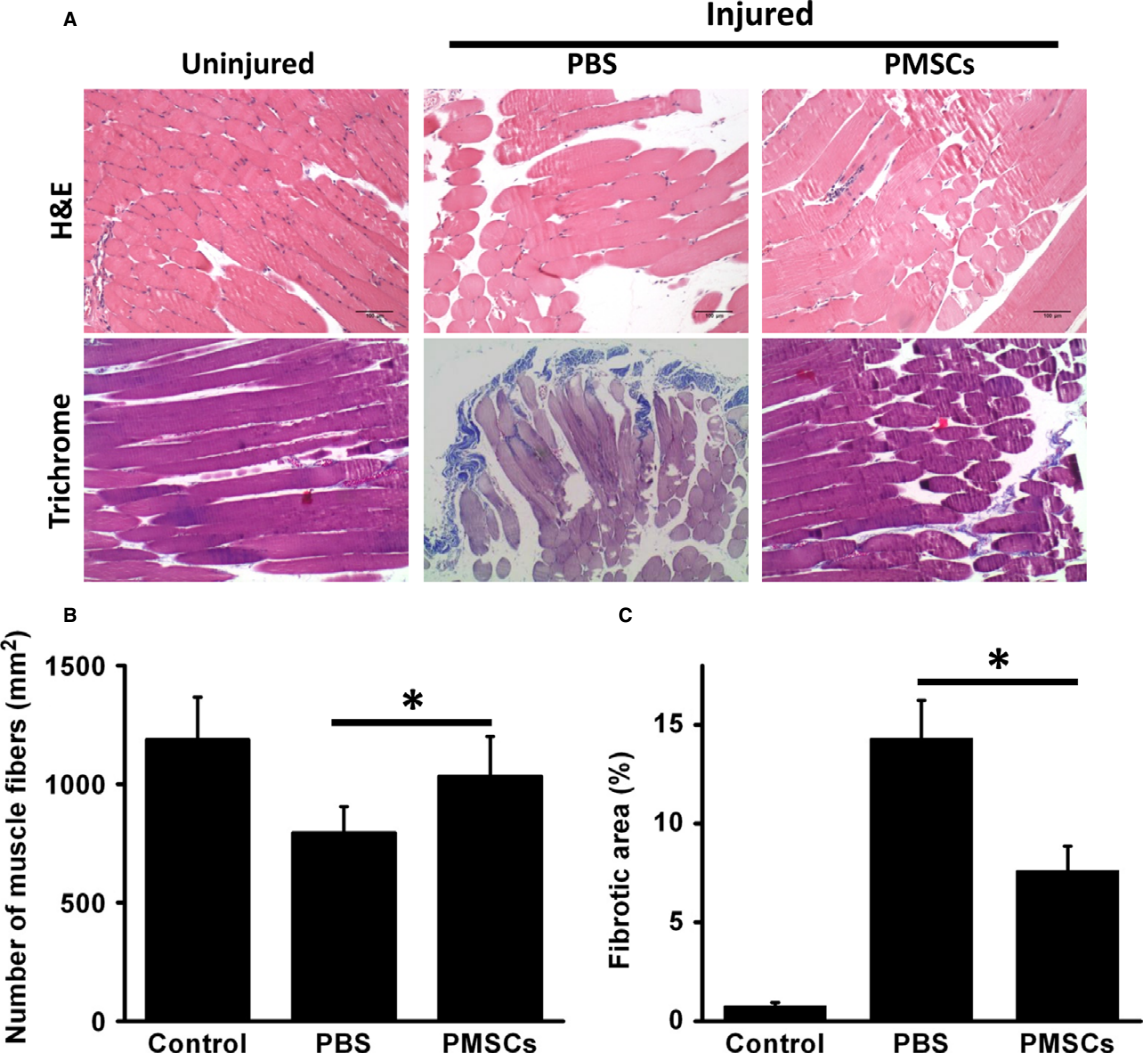
PMSCs improves pathologies associated with ischemia injury to hindlimbs. (**A**) At day 21 after treatment, hematoxylin and eosin staining for ischemic limbs showed massive muscle degeneration in the PBS group. In the PMSCs group, the muscle degeneration of limb ischemia was largely prevented. Masson's trichrome staining showed that fibrosis was obviously attenuated in PMSCs‐treated limbs. (**B**) Muscle fibre numbers per mm^2^ were quantified in order to show the regeneration rate of muscle fibre in different groups. (**C**) Fibrotic area was calculated based on Masson's trichrome staining. Data were presented by mean ± SD, *P < 0.05.

To further investigate the effects of PMSCs treatment, pathological analysis was performed. Consistent to physiological assessment, histological examination of the muscle revealed extensive muscle degeneration and pronounced interstitial fibrosis in the mice in the ischemic hindlimb group (Fig. 4A *middle panels*). In contrast, remarkably reduced fibrosis and less muscle degeneration were observed in the PMSC‐treated group (Fig. 4A, *right panels*). Compared to mice with PBS treated ischemic hindlimbs, mice in the PMSC treatment group exhibited significantly less fibrosis (Fig. 4A, *lower panels* with trichrome staining and Fig. 4C) and more muscle regeneration (Fig. 4A, *upper panels* with H&E staining and Fig. 4B).

### Transplanted PMSCs target to vasculature and contribute to angiogenesis

**Figure 5:**
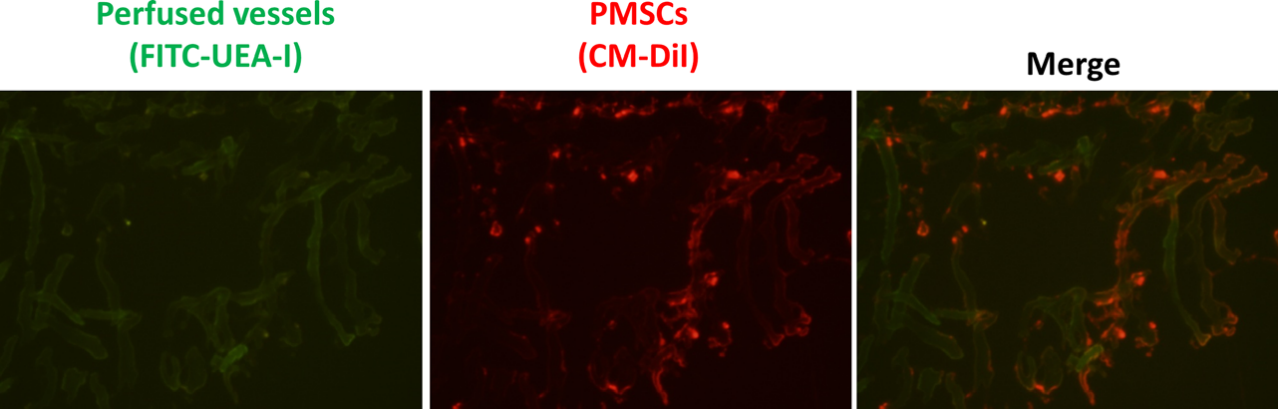
Transplanted PMSCs target to capillary network and participate in angiogenesis *in vivo*. The murine capillary network was stained with FITC‐UEA‐I (*left panel*). The CM‐DiI‐ labelled cells sprouted from local injection sites (*middle panel*). Merged image (*right panel*) shows the incorporation of placenta‐derived stem cells into murine vascular networks.

To confirm the presence of PMSCs in the recovery of ischemia injured hind limbs, PMSCs were labelled with fluorescent CM‐DiI, enabling identification of cells derived from injected PMSCs. Ischemic hindlimbs treated with labelled PMSCs were isolated and analysed by fluorescent microscopy at day 14. Before sacrificing the mice, we intravenously injected FITC‐UEA‐l with green fluorescence to enhance the contrast of perfused vessels and to test whether the vascular networks had connected to the mouse circulation. Green fluorescence identifies FITC‐UEA‐l binding endothelial cells in functional vessels, and red fluorescence indicates CM‐DiI‐labelled PMSCs. Our results suggest the formation of functional endothelial cells that successfully integrated into local tissue vascular networks of the mice (Fig. 5). Therefore, directly implicating the presence of PMSCs in the development of angiogenesis in this model.

### Differentiation ability of PMSCs in ischemic limbs

**Figure 6:**
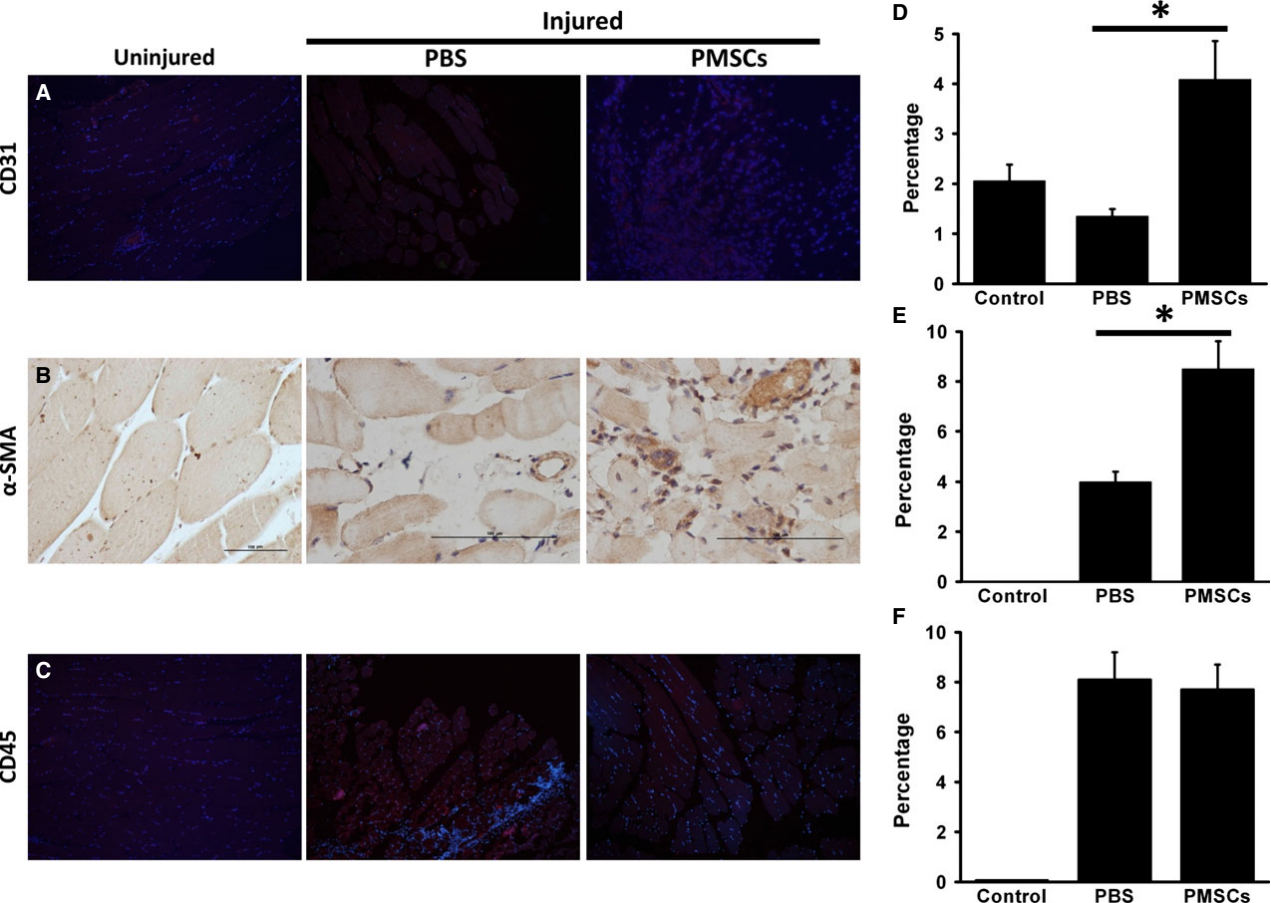
The differentiation ability of PMSCs in ischemic limbs. (**A**) Representative images of endothelial differentiation with CD31^+^ staining. (**B**) Smooth muscle differentiation with α‐SMA ^+^ staining. (**C**) Inflammation with CD45^+^ staining. Quantitative measurement was expressed as percent of positive staining *versus* total per muscle area (**D**–**F**). Data were presented by mean ± SD, *P < 0.05.

Finally, to test whether the engrafted PMSCs contribute to vascular regeneration by *de novo* differentiation, the differentiation ability of PMSCs in ischemic limbs after transplantation was determined up to day 21. Staining with anti‐human CD31 PMSCs showed that sporadic cells expressed CD31, indicating endothelial differentiation of PMSCs in the ischemic limbs (Fig. 6A and D). The improvement in angiogenesis was further supported by α‐SMA expression analysis. Immunostaining showed that anti‐human α‐SMA cells were detected in PMSC‐treated tissues after 21 days. α‐SMA^+^ cells were found in small arteries, suggesting that PMSCs contributed to arteriole formation (Fig. 6B and E). Some cells were also found between muscle fibres or among muscle tissues. These results indirectly demonstrate that intravascular delivery of PMSCs can lead to limb neovascularization. PMSC injection as a treatment for hind limb ischemia did not cause massive inflammation as demonstrated by CD45 staining (Figs 4C and 6F).

## Discussion

In the present study, we demonstrated that PMSCs are a novel source for stem cell therapy in hind limb ischemia model. Our studies can be summarized into three major findings. First, PMSCs were capable of differentiating into endothelial and smooth muscle cells *in vivo*. Second, PMSCs were observed to target the vasculature with PMSC transplantation enhancing angiogenesis and increasing blood flow and capillary density in the ischemic hind limb. Finally, the therapeutic effects of PMSCs may be related to their ability to differentiate and contribute to angiogenesis in ischemia injured hind limbs.

Mesenchymal stem cell‐based angiogenesis therapy has emerged as one of the most promising therapeutic strategies for the treatment of PAD. While MSCs are widely distributed in a variety of tissues in the adult human body (*e.g*., bone marrow 17, fat tissue 18, lung 19, and liver 13), they are present in a low abundance in these tissues. Even in the most well‐studied and accessible source of MSCs, bone marrow, it has been estimated that MSCs represent only 0.001–0.01% of the total nucleated cells 14. Thus, there is an imbalance between the demand for and the vailability of MSCs for therapeutic applications.

Our data, along with previous researchers', illustrate that viable MSCs are also present in human term placenta 9, 15. Compared with conventional adult MSCs, PMSCs have several advantages. First, placentas are abundantly available from maternity wards but are often discarded, making them an ideal source of MSCs for multiple applications. Second, no invasive procedures are required to obtain placental MSCs, unlike BM‐MSCs. PMSCs can be easily isolated and expanded without morphological and characteristic changes. Third, clonally expandable PMSCs share many characteristic markers with human BM‐MSCs, such as CD29, CD13, CD73, CD49b 16, 20, but they appear different from previously described populations of adult stem cells, multipotent adult progenitor cells, or clonally isolated human BM multipotent stem cells. In particular, PMSCs expressed significantly higher amounts of CD10 and CD49d and were negative for CD34 and CD45, compared to BM‐MSCs, which is in line with earlier reports 14, 21, 22. Fourth, we noted that PMSCs not only preserved the phenotypes of MSCs but also exhibited the genetic characteristics of embryonic stem cells (ESCs). PMSCs expressed markers of ESCs, including OCT‐4 and stage‐specific embryonic antigen SSEA‐4, which are believed to be essential for the regulation of differentiation 23, 24. Finally, our current study demonstrated that PMSC injection as a treatment for hindlimb ischemia enhances angiogenesis, suggesting that PMSCs are a good candidate as a novel source of stem cell therapy for the treatment of arterial disease.

Restoring blood flow to the site of injured tissue is a prerequisite for mounting a successful repair response. Studies have confirmed that engraftment of MSCs facilitates collateral blood flow in ischemic regions, but the mechanism by which MSCs contribute to new vessels remains controversial. It has been reported that MSCs from different tissues (*e.g*., adipose tissue 25, bone marrow 26, umbilical cord 27, and skin 28) can differentiate into vascular endothelial cells. In our previous study, we also demonstrated that PMSCs acquire endothelial‐like characteristics when cultured in endothelial growth supplement 9. These characteristics include cord formation in response to extracellular matrix and expression of endothelial markers. The ability to form new capillary‐like structures upon plating on basement matrix gel suggests that differentiated PMSCs have the potential to participate in angiogenesis. However, direct *in vivo* evidence for the contribution of MSC‐derived endothelial cells to new vessel formation is lacking. In the present study, we evaluated whether PMSCs were able to form functional blood vessels *in vivo*. We found that Cord‐like networks developed from PMSCs were observed as early as 4 days after implantation. To test whether the vascular network connected to the mouse circulatory system, we intravenously injected FITC‐ labelled UEA‐I to enhance the contrast of perfused vessels (Fig. 3). Interestingly, we found that transplanted PMSCs localized to these perfused vessels, indicating PMSCs might directly contribute to angiogenesis in ischemia injured hindlimbs. In addition to recover blood flow, we also demonstrated that PMSCs treatment could notably reduce limb necrosis and autoamputation (Fig. 1), indicating newly formed blood vessels participate in regeneration of ischemic injured tissues.

One of the concerns for our study is the potential rejection after human PMSCs were injected into immunocompetant mice. Interestingly, human MSCs have been used to multiple species, including mice 29, 30, rats 31, rabbits 32 and dogs 33 in different disease settings, such as dermal wound, spinal cord injury and neurodegeneration diseases. Consistent with these studies, we did not observe obvious rejection responses after human PMSCs were transplanted into mice. Although the molecular mechanisms for these observations are still largely unknown, our data did provide evidence to show the low immunogenicity of human PMSCs. Another issue is how is the average cell survival rate after implantation? Although our data in Figure 5 suggested that the transplanted PMSCs can survival and incorporate into blood vessel, we do not know how many of the injected PMSCs finally survived and functioned. This is a hot research direction in stem cell‐based therapy, because the survival rate determines the potential efficacy of the stem cell treatment (or the reason for the failure of stem cell‐based therapy). Currently, the best non‐invasive way is a bioluminescence based imaging to quantify live stem cells *in vivo* 34, which will be our future research study direction. Finally, more studies are required to characterize the detailed actions for PMSCs on angiogenesis process. We will perform more experiments at different time points following PMSCs implantation to dissect the cellular and molecular pathways which are involved in PMSCs‐mediated angiogenesis.

There are several challenges in MSCs‐based therapy. First, it has been previously described that transplanted MSCs are sensitive to ischemic and inflammatory microenvironments 35. In the present study, PMSCs treatment did not cause massive rejection (minimal inflammatory response was observed in our pathology slides), indicating that *in vivo* PMSCs therapy may be a source of cells with less immunogenicity as compared to other MSCs. Second, cell viability after transplantation is extremely low 36, it has been shown that more than 90% MSCs will die within 7 days of transplantation. We are excited in our present observations that PMSCs persisted for 28 days, which is long enough for angiogenesis to take place.

As PMSCs‐base therapy is a complex regenerative process, our current study cannot cover every aspect of the beneficial effects of PMSCs treatment. For example, our previous studies found that the paracrine factors secreted by MSCs might contribute to the angiogenesis in multiple animal models 9, 37. Our future studies will focus on identifying the paracrine factors that are critical in angiogenesis in limb ischemia. In addition to paracrine secretion, MSCs may also play a role in immunomodulation. A central question will be: do PMSCs protect limb against ischemia injury by suppression of the initial inflammatory response? Finally, although we can successfully isolate PMSCs for our preclinical studies 9, reproducible and efficient methods to isolate, expand, and deliver PMSCs for clinical trial are needed. This might require new technologies as well as more regulatory oversight in human applications.

## Conflicts of interest

The authors confirm that there are no conflicts of interest.
